# Immunomodulatory crosstalk between GPCR and hippo signaling in cancer: implications for tumor immunity and therapeutic targeting

**DOI:** 10.3389/fimmu.2026.1765361

**Published:** 2026-05-11

**Authors:** Xiangxiang Ren, Tianhao Xie, Litao Liu, Xiaoshi Jin, Meng Zhang

**Affiliations:** 1 Department of General Surgery, Affiliated Hospital of Hebei University, Baoding, Hebei, China; 2 Department of Dermatology, Affiliated Hospital of Hebei University, Baoding, Hebei, China

**Keywords:** GPCR (G protein-coupled receptors), hippo signaling pathway, immunotherapy targeting, tumor immune microenvironment, YAP/TAZ

## Abstract

G protein-coupled receptors (GPCRs) and the Hippo signaling pathway are central regulators of tissue homeostasis, cell proliferation, and immune modulation. Emerging evidence reveals a pivotal, bidirectional crosstalk between these systems in cancer initiation, progression, and immune evasion. This review systematically outlines the molecular mechanisms of GPCR--Hippo interactions, with a particular emphasis on their immunomodulatory functions within the tumor microenvironment (TME). We highlight how distinct G protein subtypes (e.g., Gs vs. G12/13) exert opposing effects on the core Hippo kinase cascade, thereby modulating the activity of the downstream transcriptional co-activators YAP/TAZ. These effectors are key regulators of immune checkpoint expression (e.g., PD-L1), stromal reprogramming, and immune cell recruitment/function. Notably, oncogenic positive-feedback transcriptional loops (e.g., OXTR--YAP, CXCR7--YAP) have been identified as potent self-reinforcing drivers of both malignancy and immunosuppression. Furthermore, biased GPCR signaling and integration with other pathways (e.g., Wnt, TGF-β) form a context-dependent regulatory network that shapes innate and adaptive antitumor immunity. Therapeutically, this axis offers a rich landscape for intervention, including direct YAP/TAZ--TEAD inhibitors, selective GPCR modulators, drug repurposing, and rational combinations with immune checkpoint blockade (ICB). Biomarkers based on GPCR expression, Hippo activity, and immune cell spatial signatures are critical for patient stratification. Future research should leverage single-cell multi-omics, spatial biology, and machine learning to decipher cell-specific signaling within the TME and accelerate the translation of immune-modulating combinatorial therapies.

## 1 Introduction: fundamental characteristics of GPCR and hippo pathways and their association with cancer

### 1.1 Overview of the GPCR signaling system

G protein-coupled receptors (GPCRs) constitute the largest family of membrane proteins in the human genome, mediating cellular responses to diverse extracellular signals such as hormones, neurotransmitters, chemokines, and lipids (1, 2). Canonical signaling is transduced via heterotrimeric G proteins and scaffold proteins like β-arrestin (3). The concept of “biased signaling,” where ligands preferentially activate G protein- or β-arrestin-dependent pathways, has advanced precision drug design (4). In cancer, GPCRs contribute to multiple hallmarks of tumorigenesis, including proliferation, invasion, metastasis, and immune microenvironment remodeling (5, 6). Many GPCRs are expressed on immune cells and cancer-associated fibroblasts (CAFs), influencing cytokine secretion, immune cell recruitment, and checkpoint expression (6), positioning them as central players in intercellular communication within the TME.

### 1.2 Core components and regulatory mechanisms of the hippo signaling pathway

The Hippo pathway is an evolutionarily conserved kinase cascade that critically regulates organ size, tissue homeostasis, and immune surveillance (7). Its core components in mammals include the upstream kinases MST1/2 and LATS1/2, and the downstream transcriptional co-activators YAP and TAZ. When active, MST1/2 phosphorylates and activates LATS1/2, which then phosphorylates YAP/TAZ, leading to their cytoplasmic retention and degradation (8). Conversely, pathway inactivation results in dephosphorylated YAP/TAZ translocating into the nucleus. There, they bind TEAD family transcription factors to drive expression of genes promoting cell proliferation, survival, stemness, and immunomodulatory factors such as PD-L1 (8–10). The pathway integrates diverse inputs including cell density, mechanical stress, energy status, and GPCR signaling (7). Dysregulation, particularly YAP/TAZ hyperactivation, is strongly implicated in tumorigenesis, metastasis, therapy resistance, and immune evasion (9, 11, 12).

### 1.3 Research background and significance

The foundational link between GPCR and Hippo pathways was established by Yu et al. (2012), who demonstrated that lysophosphatidic acid (LPA) and sphingosine-1-phosphate (S1P) inhibit LATS1/2 via G12/13-coupled receptors to activate YAP/TAZ, whereas glucagon activates LATS1/2 and inhibits YAP through Gs-coupled receptors (13, 14). This work positioned Hippo--YAP as a key downstream effector of GPCR signaling. Building upon this, emerging evidence suggests that the GPCR--Hippo network functions as a context-dependent signaling module capable of actively driving cancer progression and shaping the immunosuppressive TME. This occurs through positive-feedback loops and direct regulation of immune cell function, thereby representing a promising therapeutic target system for combination immunotherapy. This review aims to systematically elucidate these mechanisms with an immunological focus, integrate insights into immune metabolism and cellular crosstalk, and evaluate translational opportunities for precision oncology.

## 2 Molecular mechanisms of GPCR--hippo pathway crosstalk

### 2.1 G protein-dependent regulation of the hippo kinase cascade

GPCRs modulate the core Hippo kinases in a highly context-sensitive manner that is dictated by their coupling to distinct G protein subtypes (13). Gs-coupled Receptors, such as the glucagon receptor, typically activate the Hippo pathway. Upon ligand binding, they stimulate adenylyl cyclase, elevating intracellular cAMP levels and activating protein kinase A (PKA). The cAMP-PKA axis subsequently promotes LATS1/2 kinase activity, leading to increased YAP/TAZ phosphorylation, cytoplasmic retention, and functional inhibition (13, 14). This pathway may also influence immune cell function within the TME, as cAMP is a well-known immunomodulator. In contrast, G12/13- and Gq/11-coupled Receptors, which respond to ligands like lysophosphatidic acid (LPA) and sphingosine-1-phosphate (S1P), generally inhibit the Hippo pathway. Their activation engages Rho GTPases, which in turn modulate actin cytoskeleton dynamics. These cytoskeletal rearrangements directly inhibit LATS1/2 activity, resulting in the dephosphorylation and nuclear translocation of YAP/TAZ (13, 14). The transcriptional programs initiated by activated YAP/TAZ can subsequently drive the expression of cytokines and chemokines that recruit immunosuppressive cells into the TME. Adding further complexity, the outcome of this regulation can vary significantly based on cellular context. Furthermore, the phenomenon of biased signaling, where a ligand preferentially activates G protein- or β-arrestin-dependent pathways downstream of the same GPCR, offers an additional layer of control over Hippo pathway modulation and its subsequent immune outcomes (4).

### 2.2 β-arrestin-mediated modulation of hippo signaling

Beyond canonical G protein-dependent signaling, β-arrestins—traditionally recognized for their role in GPCR desensitization and internalization—function as critical signaling scaffolds that modulate Hippo pathway activity in a G protein-independent manner. β-arrestins (primarily β-arrestin-1 and β-arrestin-2) bind to activated GPCRs and recruit a diverse array of signaling molecules, including Src family kinases, mitogen-activated protein kinases (MAPKs), and E3 ubiquitin ligases, thereby initiating distinct signaling cascades that can converge on the Hippo core kinases or directly on YAP/TAZ (4, 15).

A well-characterized example of this mechanism comes from high-grade serous ovarian cancer, where endothelin A receptor (ETAR) activation promotes the formation of a β-arrestin-1/YAP/mutant p53 complex. This ternary complex facilitates YAP nuclear translocation and enhances its transcriptional activity independently of the classical LATS1/2 phosphorylation cascade, thereby driving tumor progression and conferring resistance to both chemotherapy and targeted agents (15). Similarly, in breast cancer, β-arrestin-1 has been shown to interact with TAZ, promoting its stabilization and nuclear accumulation in response to GPCR activation, an axis that contributes to cancer stem cell--like properties and metastatic potential (16).

Importantly, β-arrestin--mediated Hippo regulation extends beyond tumor cell--intrinsic effects to influence immune cell function within the TME. β-arrestins are expressed in various immune cell subsets, where they modulate chemokine receptor signaling, migration, and cytokine production. For instance, β-arrestin-2 regulates CXCR4-mediated migration of T cells and dendritic cells (17), and its involvement in YAP/TAZ signaling in immune cells—though still poorly understood—may represent an unexplored node of crosstalk between GPCR signaling and immune effector function. Moreover, β-arrestin--dependent signaling has been implicated in the regulation of PD-L1 expression in certain tumor types, suggesting a potential link to immune checkpoint regulation that warrants further investigation.

From a therapeutic perspective, the β-arrestin--YAP/TAZ axis offers unique opportunities for intervention. The development of β-arrestin--biased ligands may enable more precise modulation of Hippo signaling in cancer, potentially uncoupling desired antitumor effects from on-target toxicities associated with global pathway inhibition. Additionally, targeting downstream effectors of β-arrestin--YAP/TAZ signaling, such as the YAP--TEAD interaction, may circumvent the complexity of upstream receptor heterogeneity and provide a more universal strategy to disrupt this oncogenic axis (18). Collectively, these findings establish β-arrestins as central, G protein-independent nodes in the GPCR--Hippo signaling network.

### 2.3 Bidirectional feedback between YAP/TAZ and GPCR signaling

YAP/TAZ actively engage in reciprocal regulation of GPCR expression and function, establishing potent feedback loops that can amplify oncogenic and immunosuppressive programs (19).

YAP/TAZ directly promote the transcription of several GPCR genes. In gastric cancer, YAP binds the *CXCR7* promoter to enhance its expression; conversely, CXCR7 signaling via Gαq/11-Rho activates YAP, forming a CXCR7--YAP positive-feedback loop associated with specific immune cell infiltration patterns (20). Similarly, in hepatocellular carcinoma (HCC), YAP drives *OXTR* expression, while OXTR signaling inhibits LATS1/2 via Gαq/11-ROCK, establishing an oncogenic OXTR--YAP axis (21). In addition to transcriptional control, YAP/TAZ may influence GPCR signaling output indirectly by modulating chromatin accessibility, cytoskeletal organization, and cellular metabolism, processes that also affect immune cell communication (14).

### 2.4 Differential regulatory patterns across GPCR families

Different GPCR families exhibit distinct patterns of Hippo pathway regulation, presenting opportunities for subtype-specific intervention and immune modulation. These divergent effects, which range from YAP/TAZ activation to inhibition depending on the primary G protein coupling, are systematically summarized in **Table 1**.

**Table 1:** Selected GPCRs modulating the hippo pathway and immune responses in cancer.

GPCR subfamily/receptor	Primary G protein coupling	Effect on pippo/YAP	Cancer context	Key immune modulatory role & proposed mechanism	Associated immune phenotype/clinical correlation
LPAR1-3	G12/13, Gq/11	Inhibits LATS, Activates YAP/TAZ	Multiple (e.g., breast, ovarian)	Promotes TAM recruitment & M2 polarization; enhances cytokine/chemokine (e.g., CCL2, IL-6) secretion.	Immunosuppressive TME; correlates with poor ICI response in models.
LPAR6	Gi/o, Gs	Inhibits YAP/TAZ nuclear translocation	Hepatocellular Carcinoma	Potential role in modulating Kupffer cell activity; context-dependent, may have tumor-suppressive effects.	Less defined; possibly altered intrahepatic immune surveillance.
CXCR7	Gq/11, β-arrestin	Activates YAP (via Rho), forms feedback loop	Gastric Cancer	Drives immunosuppression: Associated with increased Tregs, M2 macrophages, MDSC infiltration, and PD-L1 upregulation (20).	“Immune-cold” phenotype; potential biomarker for ICI resistance.
OXTR	Gq/11	Inhibits LATS (via ROCK), activates YAP	Hepatocellular Carcinoma	Linked to immune evasion; core of oncogenic feedback loop; may regulate intratumoral immune cell composition (21).	Contributes to immunosuppressive microenvironment; OXTR antagonism shows preclinical efficacy.
S1PR1-5	Gi, G12/13, Gq (varies)	Activates YAP/TAZ (via Gi/Rho)	Multiple, including Lymphoma, solid tumors	Critical for immune cell egress & function: Regulates lymphocyte trafficking, DC migration, and TAM polarization.	Modulates immune cell access to tumors; S1PR modulators in clinical use.
GPRC5A	Unknown	Transcriptional upregulation of YAP1	NSCLC, Pancreatic Cancer	May influence tumor immunogenicity; loss associated with CD8+ T cell exclusion in NSCLC (52, 53).	Correlates with “immune-cold” status; potential predictive biomarker.
TGR5 (GPBAR1)	Gs	Modulates miR-139-5p/DDIT4 axis	Cervical Cancer, others	May attenuate inflammatory signaling in the TME; expressed on immune cells (e.g., macrophages).	Potential link to metabolic-inflammation axis in TME (54).

### 2.5 Integration with other signaling pathways and cellular cues

**Figure 1:**
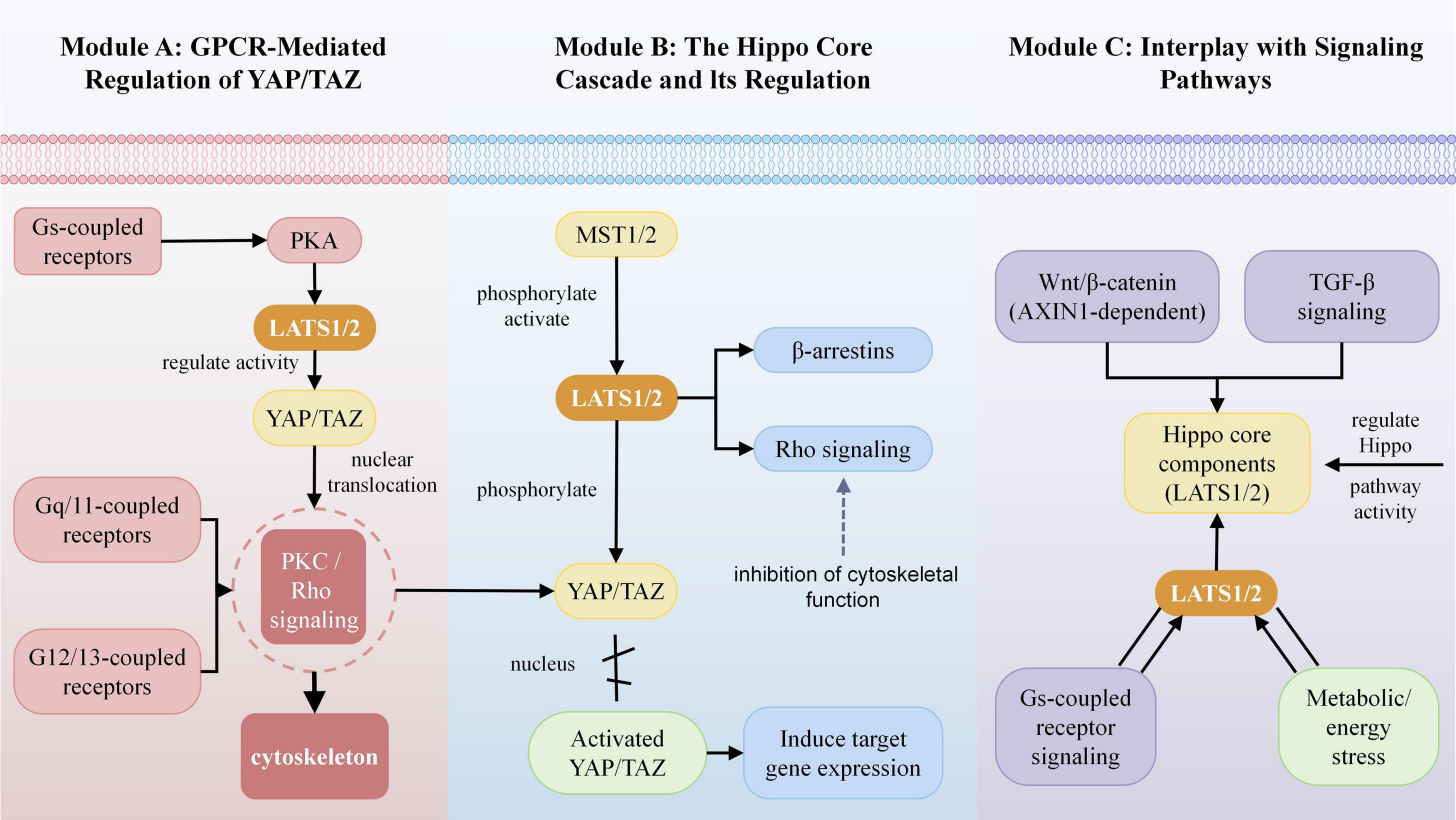
Molecular crosstalk between GPCR signaling and the hippo pathway. **(A)** GPCR-mediated regulation of YAP/TAZ. Gs-coupled receptors modulate YAP/TAZ activity, potentially through pathways involving PKA activation. Gq/11- and G12/13-coupled receptors influence cytoskeletal dynamics and YAP/TAZ nuclear translocation through signaling regulators such as RhoA. **(B)** The hippo core cascade and its regulation. The core hippo kinase cascade: MST1/2 phosphorylates and activates LATS1/2, which in turn phosphorylates YAP/TAZ, leading to their cytoplasmic retention and functional inhibition. β-arrestins and rho signaling are implicated in the regulation of YAP/TAZ activity. Activated YAP/TAZ translocate to the nucleus to induce target gene expression. **(C)** Interplay with other signaling pathways. Crosstalk with the Wnt/β-catenin pathway (e.g., via AXIN1) and other hippo components. TGF-β signaling can influence the activity of the Hippo pathway. Gs-coupled receptor signaling and metabolic or energy stress are indicated as regulators of LATS1 activity.

The GPCR--Hippo axis is embedded within a broader signaling network that includes key immune-related pathways, as illustrated in **Figure 1**. Crosstalk with Wnt/β-catenin is a prime example. In HCC, *AXIN1* mutations create a permissive context for enhanced YAP/TAZ signaling (22, 23). This interplay may also affect T cell activity, as Wnt/β-catenin signaling in tumor cells can exclude CD8+ T cells, thereby contributing to an immune-cold phenotype. Similarly, the interaction with TGF-β, a master regulator of immune suppression, suggests potential tripartite crosstalk. TGF-β signaling can downregulate specific Gs-coupled receptors, potentially altering the cellular “GPCR landscape” and its effect on Hippo regulation (24, 25). This highlights how changes in the cytokine milieu can rewire how tumor cells respond to GPCR ligands. Metabolic and stress integration also plays a role; nutrient-sensing GPCRs and energy stress sensors like AMPK intersect with Hippo signaling. Under energy deprivation, AMPK can phosphorylate and activate LATS1, leading to YAP/TAZ inhibition (26, 27). This suggests that metabolic reprogramming in the TME, including in immune cells, can dynamically influence the GPCR-Hippo axis.

### 2.6 Cytoskeletal architecture as an independent regulator of hippo signaling

In addition to the signaling pathways discussed above, the integrity of the cytoskeleton itself can regulate Hippo pathway activity independently of GPCR signaling. The cytoskeleton not only acts as a downstream effector of GPCRs in mechanotransduction to YAP/TAZ, but its own components can directly modulate pathway activity through physical interactions with core Hippo kinases. A recent study in hepatocellular carcinoma (HCC) has revealed a novel mechanism underlying this regulatory mode: the cytoskeletal protein PDZ and LIM domain protein 1 (PDLIM1) competitively binds to the F-actin crosslinking protein α-actinin 4 (ACTN4), thereby restricting excessive F-actin polymerization, promoting LATS1 kinase phosphorylation, and suppressing YAP nuclear translocation and transcriptional activity (28). This study further demonstrated that the interaction between PDLIM1 and ACTN4 depends on the asparagine 145 (Asn145) residue of PDLIM1, and mutation of this site completely abolishes the inhibitory effect of PDLIM1 on F-actin polymerization and its activating function on Hippo signaling (28). Notably, excessive F-actin polymerization induced by PDLIM1 deficiency significantly enhances the binding of LATS1 to F-actin, subsequently inhibiting LATS1 phosphorylation and leading to YAP activation (28). This mechanism reveals an independent regulatory function of cytoskeletal components as an upstream “brake” on Hippo signaling (28), which may act synergistically with or complement the GPCR--Rho--actin regulatory axis to collectively determine the activation status of YAP/TAZ.

## 3 The GPCR–hippo axis as a master regulator of the tumor immune microenvironment

The GPCR–Hippo axis functions as a central hub that integrates tumor-intrinsic signaling with extrinsic immune and stromal cues, thereby shaping the composition, functional state, and spatial architecture of the tumor immune microenvironment (TME). Emerging evidence indicates that this axis exerts its immunomodulatory effects through multiple coordinated mechanisms, ranging from direct regulation of tumor cell immunogenicity to reprogramming of myeloid cells, adaptive immune responses, and cancer-associated fibroblasts (CAFs) (29).

### 3.1 Regulation of tumor cell-intrinsic immunogenicity

Constitutively active YAP/TAZ directly transcribe genes encoding immune checkpoint ligands, most notably PD-L1, thereby facilitating immune evasion (30, 31). This transcriptional regulation is further amplified by upstream GPCR signaling, which can enhance YAP/TAZ nuclear localization and transcriptional activity. Beyond PD-L1, YAP/TAZ activation has been shown to downregulate components of the antigen presentation machinery, including MHC class I molecules, and to modulate the secretion of immunoregulatory cytokines and chemokines. Collectively, these tumor cell-intrinsic changes establish an immune-evasive contexture that limits T cell recognition and effector function.

### 3.2 Shaping the myeloid compartment: macrophages and myeloid-derived suppressor cells

The GPCR–Hippo axis plays a pivotal role in orchestrating the recruitment and functional polarization of myeloid cells within the TME. Tumor-derived ligands such as lysophosphatidic acid (LPA) and sphingosine-1-phosphate (S1P), acting through their cognate GPCRs, activate YAP/TAZ in cancer cells, leading to the upregulation of chemokines including CCL2 and CSF1, which promote monocyte recruitment (13, 32). Once recruited, these monocytes are instructed toward an immunosuppressive M2-like tumor-associated macrophage (TAM) phenotype by YAP/TAZ-driven secretion of factors such as IL-6 and VEGF. This reciprocal crosstalk is further reinforced by M2 TAMs, which secrete additional GPCR ligands (e.g., S1P), establishing a feed-forward amplification loop. Similarly, the GPCR–Hippo axis contributes to the recruitment and activation of myeloid-derived suppressor cells (MDSCs). For instance, the CXCR7–YAP feedback loop in gastric cancer is associated with increased infiltration of granulocytic MDSCs, while YAP/TAZ-driven production of prostaglandin E2 (PGE2) and reactive oxygen species (ROS) further enhances MDSC suppressive capacity (20).

### 3.3 Modulation of adaptive immune responses

The impact of the GPCR–Hippo axis on adaptive immunity is multifaceted and predominantly immunosuppressive. Cytotoxic CD8+ T cells are antagonized through at least three convergent mechanisms: YAP/TAZ-mediated upregulation of PD-L1 (33) on tumor cells directly promotes T cell exhaustion; metabolic reprogramming of the TME, including acidification and nutrient deprivation, limits T cell metabolic fitness; and the recruitment of TAMs and MDSCs establishes physical and chemical barriers that restrict T cell infiltration and function. In parallel, the axis fosters a regulatory T cell (Treg)-favorable environment by upregulating chemokines such as CCL22 and their cognate receptors (e.g., CCR4), thereby facilitating Treg recruitment into the TME and reinforcing local immunosuppression (16). Emerging evidence also suggests potential roles for this signaling network in modulating B cell function (34), tertiary lymphoid structure (TLS) formation (35), and natural killer (NK) cell activity (36), although these areas remain incompletely understood and warrant further investigation.

### 3.4 Impact on cancer-associated fibroblasts and stromal architecture

Cancer-associated fibroblasts (CAFs) function as both regulators and targets of the GPCR–Hippo axis. As a major source of GPCR ligands, including LPA and S1P, CAFs contribute to paracrine activation of YAP/TAZ in neighboring tumor cells. Conversely, GPCR signaling in CAFs themselves can activate YAP/TAZ, driving a contractile, inflammatory phenotype characterized by extracellular matrix (ECM) remodeling and secretion of immunosuppressive cytokines such as CXCL12 (13, 32). This CAF-mediated stromal reprogramming not only alters the physical barriers to immune cell infiltration but also establishes a chemokine landscape that perpetuates immune exclusion.

### 3.5 Therapeutic Reprogramming of the Immune Microenvironment

Given its central role in coordinating immunosuppressive networks, the GPCR–Hippo axis represents an attractive therapeutic node for reprogramming the TME. Inhibiting oncogenic GPCRs or the YAP/TAZ–TEAD transcriptional complex can downregulate PD-L1 expression, reduce the secretion of T cell–excluding chemokines, and diminish the recruitment of TAMs and MDSCs, thereby promoting a shift from an immune-”cold” to an immune-”hot” tumor phenotype (37). Moreover, this axis has been identified as a key mediator of adaptive resistance to immune checkpoint blockade (ICB); its inhibition can prevent the reactivation of immunosuppressive programs that typically emerge upon ICB treatment (16). These mechanistic insights provide a strong rationale for combining GPCR or Hippo pathway inhibitors with ICIs, cancer vaccines, or adoptive cell therapies, with the goal of simultaneously relieving immunosuppressive brakes and enhancing antitumor effector mechanisms (38, 39).

### 3.6 Intrinsic Roles of the GPCR–Hippo Axis in Immune Cell Function, Metabolism, and Differentiation

While the preceding sections focused on how tumor cell–intrinsic GPCR–Hippo signaling shapes the immunosuppressive tumor microenvironment (TME) in a paracrine manner, an emerging body of evidence indicates that this signaling axis also operates intrinsically within immune cells to regulate their activation, differentiation, metabolism, and effector functions. Understanding these cell-autonomous mechanisms is critical for comprehensively evaluating the therapeutic potential of targeting this pathway.

#### 3.6.1 T cells: differentiation, exhaustion, and metabolic programming

In T lymphocytes, the Hippo pathway—particularly the transcriptional co-activators YAP and TAZ—plays a context-dependent role in controlling T cell fate decisions and functional states. In naïve CD8+ T cells, YAP activity is associated with a memory precursor phenotype, whereas its suppression favors terminal effector differentiation. This balance is partly regulated by GPCR signaling, as chemokine receptors such as CXCR4 and CCR7 can modulate YAP/TAZ activity via G protein–dependent and β-arrestin–dependent mechanisms, thereby influencing T cell trafficking and long-term persistence (13, 15).

In the context of chronic antigen stimulation within the TME, YAP/TAZ activation in tumor-infiltrating T cells has been linked to the induction of exhaustion-associated genes, including inhibitory receptors such as PD-1 and TIM-3. Mechanistically, sustained GPCR signaling (e.g., through S1PR or LPAR) is known to promote YAP nuclear translocation in cancer cells and other cell types (13); whether similar mechanisms operate directly in T cells to drive exhaustion programs via cooperation with NFAT and AP-1 remains an active area of investigation, with emerging evidence suggesting such a possibility (31). Furthermore, YAP/TAZ regulate T cell metabolism by controlling the expression of key glycolytic enzymes and mitochondrial biogenesis factors (40). For instance, YAP has been shown to promote glycolysis in activated T cells (41), and its aberrant activation may contribute to the metabolic dysfunction observed in exhausted T cells within the TME (42). These findings suggest that the GPCR–Hippo axis directly influences the quality and durability of T cell–mediated antitumor responses.

#### 3.6.2 Macrophages: polarization, phagocytosis, and inflammatory tone

Macrophages express a broad repertoire of GPCRs that sense damage-associated molecular patterns (DAMPs), cytokines, and metabolites, and the Hippo pathway has emerged as a key determinant of macrophage polarization and functional plasticity. In tumor-associated macrophages (TAMs), activation of Gq/11- or G12/13-coupled receptors (e.g., LPAR, S1PR) promotes YAP/TAZ nuclear accumulation, which in turn drives expression of M2-polarization markers (e.g., *Arg1*, *Il10*, *Mrc1*) while suppressing pro-inflammatory M1-associated genes (13, 32). This YAP/TAZ-driven M2 polarization is further reinforced by feed-forward loops involving autocrine GPCR ligands such as S1P.

Conversely, activation of Gs-coupled receptors (e.g., β2-adrenergic receptors, prostaglandin EP2/EP4 receptors) in macrophages can elevate cAMP levels and has been associated with modulation of the Hippo pathway in other cell types (43), though direct evidence for LATS1/2 activation in macrophages remains limited. This signaling axis may promote an M1-like inflammatory state under certain conditions (44). This dichotomy highlights how the balance of GPCR inputs—rather than the presence or absence of Hippo signaling per se—determines macrophage functional outcomes. Beyond polarization, YAP/TAZ also regulate macrophage phagocytic capacity. In preclinical models, YAP activation in TAMs has been associated with reduced phagocytosis of tumor cells, an effect that may contribute to resistance to CD47-blockade therapies (45, 46). Thus, the GPCR–Hippo axis functions as an intrinsic rheostat that controls macrophage inflammatory tone, polarization state, and phagocytic function within the TME.

#### 3.6.3 Dendritic cells: maturation, antigen presentation, and t cell priming

Dendritic cells (DCs) are central to initiating antitumor T cell responses, and their function is subject to regulation by both GPCR and Hippo signaling. Immature DCs express a variety of chemokine receptors (e.g., CCR1, CCR5, CXCR4) that guide their migration to tumor sites, and engagement of these GPCRs can modulate YAP/TAZ activity. Upon encountering maturation stimuli (e.g., DAMPs, CD40 ligand), DCs undergo a coordinated program of activation that involves downregulation of YAP/TAZ activity, which is necessary for proper upregulation of co-stimulatory molecules (CD80, CD86) and MHC class II (14, 37).

Conversely, persistent GPCR signaling in the TME (13)—such as through S1PR or LPAR—can sustain YAP/TAZ activation in DCs (47), impairing their maturation and reducing their capacity to prime naïve T cells. This mechanism contributes to the defective antigen presentation observed in many tumors. Additionally, YAP/TAZ regulate DC metabolism by controlling the expression of genes involved in lipid metabolism (48) and oxidative phosphorylation, processes that are essential for supporting the energetic demands of antigen processing and T cell priming.

#### 3.6.4 Cross-immune-cell integration and therapeutic implications

The intrinsic roles of the GPCR–Hippo axis across different immune cell subsets are not isolated but rather interconnected. For example, YAP/TAZ activation in TAMs promotes secretion of S1P (49), which can in turn activate YAP/TAZ in T cells and DCs via S1PR signaling (50), establishing a multicellular positive-feedback loop that perpetuates global immunosuppression. Conversely, therapeutic inhibition of this axis—whether through GPCR antagonists, YAP/TAZ–TEAD inhibitors, or biased ligands—may simultaneously exert direct effects on multiple immune cell compartments, converting the TME from a suppressive to a permissive state (51).

From a translational standpoint, the cell-autonomous functions of the GPCR–Hippo axis in immune cells carry important implications. First, biomarkers for patient stratification should consider not only tumor cell YAP/TAZ activity but also its status in infiltrating immune cells, as the latter may influence response to both pathway-targeted agents and immunotherapies. Second, the design of combination regimens should account for the potential dual effects of inhibitors—both on tumor cells (reducing PD-L1, reversing CAF activation) and on immune cells (enhancing T cell function, promoting DC maturation, repolarizing TAMs). Finally, the emerging recognition of metabolic regulation by YAP/TAZ in immune cells suggests that combination with metabolic modulators (e.g., metformin, glycolysis inhibitors) may yield synergistic effects by simultaneously targeting tumor cell metabolism and immune cell function.

In summary, the GPCR–Hippo axis is not merely a tumor cell–intrinsic driver of immune evasion but also an intrinsic regulator of immune cell fate, function, and metabolism. A comprehensive understanding of these cell-autonomous mechanisms will be essential for the rational development of therapies that leverage this signaling network to enhance antitumor immunity.

## 4 Cancer-type-specific analysis with an immune perspective

The functional consequences of GPCR–Hippo crosstalk exhibit notable cancer-type specificity, shaped by distinct genetic backgrounds, tissue microenvironments, and immune landscapes. Understanding this context dependency is essential for translating pathway-targeting strategies into clinically effective immunotherapies.

### 4.1 Lung cancer

In non-small cell lung cancer (NSCLC), deletion of the tumor suppressor GPRC5A correlates with Hippo pathway activation, enhanced stemness, and reduced CD8+ T cell infiltration (52, 53). YAP/TAZ activity is directly linked to PD-L1 upregulation and immune evasion, suggesting that Hippo pathway inhibition may synergize with ICB (30, 31). Additionally, targeting the apelin–APJ system, which upregulates YAP1, could simultaneously suppress epithelial-mesenchymal transition (EMT) and reverse immunosuppressive features in the TME (54).

### 4.2 Hepatocellular carcinoma

In hepatocellular carcinoma (HCC), the OXTR–YAP positive-feedback loop drives tumor proliferation and contributes to an immunosuppressive microenvironment. Preclinical studies demonstrate that the OXTR antagonist atosiban disrupts this loop and inhibits HCC growth, potentially restoring immune surveillance (21). Furthermore, *AXIN1* mutations, frequently observed in HCC, create a permissive state for YAP/TAZ activation (22, 23) offering a genetic context that may be exploited for combination therapy with immunomodulatory agents.

Hepatocellular carcinoma is one of the tumor types in which the Hippo signaling pathway has been most extensively studied and YAP/TAZ hyperactivation is most commonly observed. In addition to the OXTR–YAP positive-feedback loop described above, recent studies have further revealed the regulatory role of cytoskeletal proteins in Hippo signaling in HCC and their clinical significance. PDLIM1 is significantly downregulated in metastatic HCC tissues, and its expression level positively correlates with both overall survival and disease-free survival, suggesting its potential as a metastasis suppressor in HCC (28). Mechanistically, PDLIM1 deficiency leads to excessive F-actin polymerization, which in turn inhibits LATS1 phosphorylation, activates YAP and its downstream target genes (e.g., CTGF, CYR61) (28, 55), and induces the expression of epithelial-to-mesenchymal transition (EMT) markers (e.g., upregulation of Vimentin, downregulation of E-cadherin), ultimately promoting HCC cell migration, invasion, and lung metastasis *in vivo* (28). Notably, PDLIM1 functions as a tumor suppressor in HCC, yet has been reported to exhibit oncogenic activity in glioblastoma (56) and breast cancer (57), suggesting that this tissue-dependent functional divergence may be attributed to differences in its binding patterns with ACTN family members across distinct cell types. These findings not only further confirm the central role of YAP in HCC progression but also provide a new theoretical basis for therapeutic strategies targeting upstream cytoskeletal regulators of YAP.

### 4.3 Gastrointestinal cancers

In gastric cancer, the CXCR7–YAP positive-feedback loop serves as a key driver of tumor progression and concurrently establishes an immunosuppressive tumor microenvironment (TME). Mechanistically, activation of this loop promotes the recruitment and polarization of immunosuppressive cell populations, including regulatory T cells (Tregs), M2-polarized tumor-associated macrophages (TAMs), and myeloid-derived suppressor cells (MDSCs), thereby facilitating immune evasion and diminishing the efficacy of T cell–mediated antitumor responses (20). Beyond its role in shaping the immune landscape, the activity of this feedback loop has been linked to the expression of immune checkpoint molecules and chemokines that further reinforce immune exclusion. Importantly, molecular signatures derived from Hippo pathway–related genes have demonstrated prognostic value and, when integrated with immune infiltration patterns, may serve as predictive biomarkers for immunotherapy response (58). These findings underscore the translational potential of pathway-based stratification strategies in gastric cancer, where combining GPCR–Hippo axis inhibition with immune checkpoint blockade could represent a rational therapeutic approach.

### 4.4 Other cancer types

In breast cancer, GPCRs such as GPER and GPR141 promote tumor progression and chemoresistance through mechanisms intertwined with immune evasion, although their specific roles in modulating CAF–immune cell crosstalk remain to be fully elucidated (59, 60). In prostate cancer, stromal-derived tenascin-C (TNC) inhibits YAP/TAZ via α5β1 integrin yet paradoxically promotes metastasis, underscoring the complex, context-dependent functions of Hippo signaling within the stroma–immune axis (61). In head and neck squamous cell carcinoma (HNSCC), constitutive YAP/TAZ activation resulting from *FAT1* inactivation is a hallmark associated with immune-cold phenotypes, positioning this pathway as a prime target for immune-combination strategies (62).

## 5 Therapeutic implications, biomarkers, and clinical translation

### 5.1 Clinical rationale for targeting the GPCR–hippo–immune axis

The GPCR–Hippo axis fulfills all criteria for a clinically actionable target in oncology: it drives tumor progression and metastasis, mediates immune evasion, and contributes to resistance against established immunotherapies (21, 30). Constitutive YAP/TAZ activity has been directly linked to resistance to PD-1/PD-L1 blockade, and pharmacological intervention against this axis presents a rational strategy to synergize with existing immunotherapies, particularly in patients with specific molecular and immune profiles (31).

### 5.2 Core therapeutic development strategies

**Figure 2:**
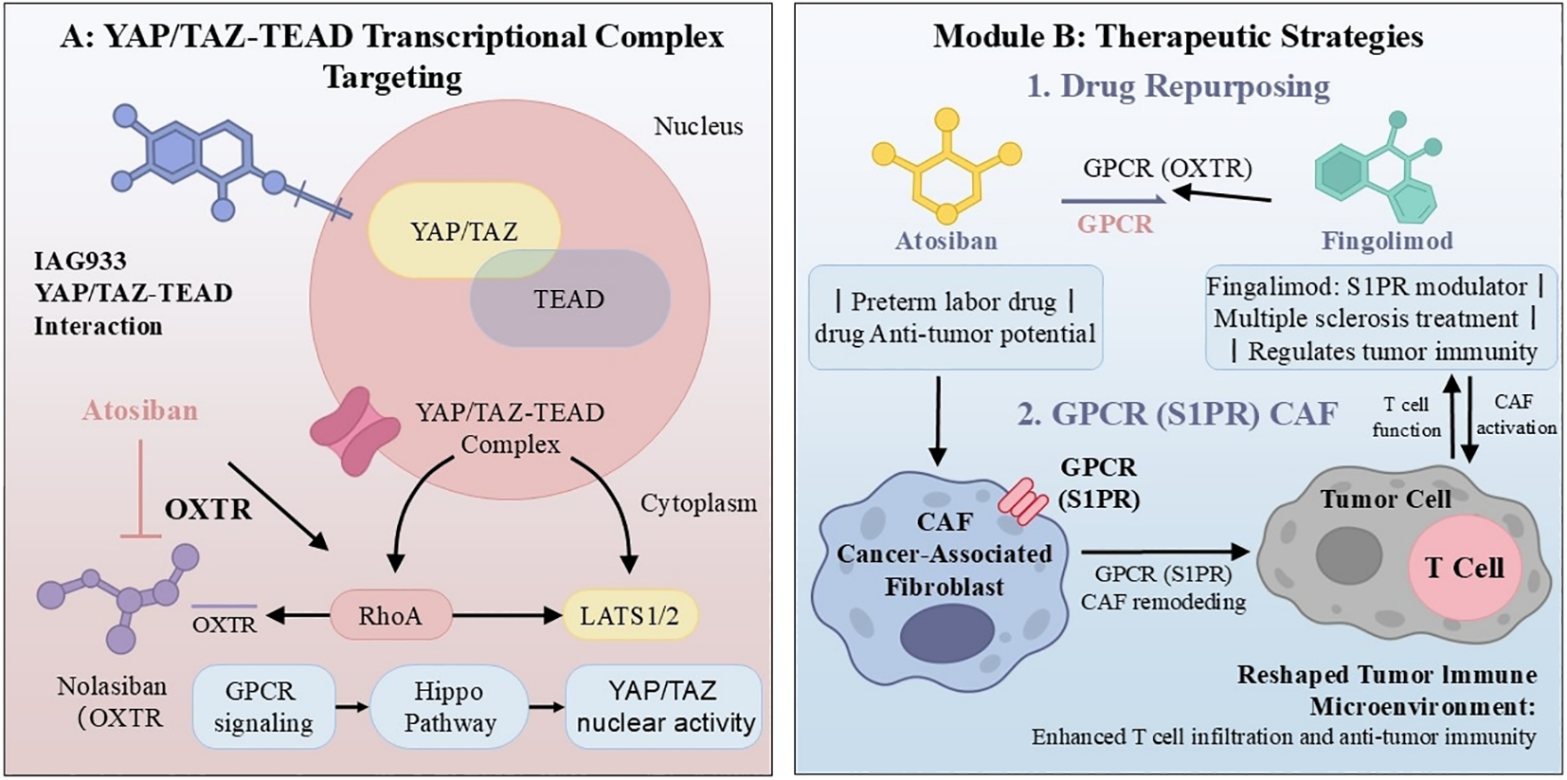
Therapeutic reprogramming of the tumor immune microenvironment by targeting the GPCR–hippo axis. **(A)** Targeting the YAP/TAZ–TEAD transcriptional complex. IAG933: An inhibitor of the YAP/TAZ–TEAD protein-protein interaction. Atosiban: An oxytocin receptor (OXTR) antagonist that modulates the Hippo pathway via a GPCR-mediated mechanism. **(B)** Therapeutic intervention strategies. *Drug repurposing approach.* Atosiban: Approved for preterm labor, with emerging antitumor applications. Fingolimod: An S1PR modulator used in multiple sclerosis, with potential effects on the tumor immune microenvironment. *Targeting cancer-associated fibroblasts (CAFs).* Modulation of CAF activity through GPCRs (e.g., S1PR) to reshape the tumor immune microenvironment.

Several complementary strategies are currently being pursued to target this axis. Direct inhibition of the YAP/TAZ–TEAD transcriptional complex using small molecules such as IAG933 and VT-3989 disrupts both the oncogenic and immunosuppressive transcriptional programs driven by these co-activators (**Figure 2**). Alternatively, upstream GPCRs can be targeted through antagonists against oncogenic receptors (e.g., OXTR) or agonists against tumor-suppressive receptors. Drug repurposing offers an accelerated path to clinical evaluation, as exemplified by atosiban (an OXTR antagonist) for HCC and S1PR modulators for solid tumors (21). Biased ligand design represents a more refined approach, aiming to selectively activate beneficial G protein pathways (e.g., Gs/cAMP) while blocking detrimental ones (e.g., Gq/Rho-mediated immunosuppression) (63). Emerging modalities such as bispecific antibodies and PROteolysis-TArgeting Chimeras (PROTACs) offer additional opportunities to couple signal inhibition with direct immune activation or to degrade key nodes like TAZ (**Table 2**) (63).

**Table 2:** Key clinical-stage agents targeting the GPCR--hippo pathway with immune combination potential.

Agent	Target/mechanism	Clinical stage	Primary indication(s) under study	Immune combination rationale & potential	Example NCT identifier/status
IAG933	YAP/TAZ--TEAD Protein-Protein Interaction Inhibitor	Phase I/II	Mesothelioma, NF2-mutant tumors, YAP/TAZ fusion tumors	High; Aims to reverse YAP/TAZ-mediated immune suppression (e.g., PD-L1 upregulation, MDSC recruitment). Rationale for combo with anti-PD-(L)1	NCT04857372 (73)
VT-3989	TEAD Palmitoylation Inhibitor	Phase I	NF2-mutant solid tumors, Malignant Pleural Mesothelioma	High; Targeting TEAD may downregulate multiple immunosuppressive genes. Synergy with ICIs expected	NCT04665206 (74)
Atosiban	OXTR Antagonist (Repurposing)	Preclinical/Planned for Exploratory	OXTR-high HCC	To be explored; Disrupts oncogenic OXTR--YAP loop. May modulate intratumoral immune cell composition (e.g., reduce Tregs) based on preclinical findings	Yang et al., Cancer Res. 2025 (21)
Fingolimod (FTY720)	S1PR Modulator (Functional antagonist)	Approved (MS), Repurposing in Oncology	Various solid tumors (investigational combinations with ICIs)	Established immunomodulator; Alters lymphocyte trafficking. May enhance ICI efficacy by sequestering Tregs in lymph nodes or affecting tumor cells/CAFs	NCT03941743 (Phase I, breast cancer) (75)

### 5.3 Rational combination strategies based on immune phenotype

The design of rational combination regimens requires careful consideration of the baseline immune phenotype and the predominant mechanisms of resistance. In immune-cold tumors characterized by sparse T cell infiltration and an immunosuppressive stroma, combining GPCR–Hippo axis inhibitors with immune checkpoint inhibitors (ICIs) and agents that promote dendritic cell maturation or T cell priming—such as cancer vaccines or STING agonists—may help initiate an antitumor immune cycle and convert “cold” tumors into “hot” ones (64, 65). In the context of acquired resistance to ICIs, where tumors initially respond but later progress, targeting specific oncogenic feedback loops such as the OXTR–YAP axis in hepatocellular carcinoma has been shown to dismantle adaptive resistance mechanisms and resensitize tumors to ICIs (66). This strategy is particularly compelling given that interferon-γ, a key effector cytokine in antitumor immunity, can induce YAP phase separation and thereby contribute to ICI resistance (66). More broadly, given the multifactorial nature of immunosuppression, combining GPCR–Hippo targeting with metabolic modulators (e.g., metformin), epigenetic drugs, or anti-angiogenic agents may enable simultaneous blockade of multiple immunosuppressive axes within the TME (67, 68). Collectively, these combinatorial approaches hold promise for achieving durable responses in patients with complex, therapy-resistant tumor ecosystems.

### 5.4 Biomarker development: integrating immune and pathway signatures

Precision deployment of GPCR–Hippo–targeted therapies hinges on the development of robust, clinically deployable biomarkers, which can be broadly categorized into three complementary groups. Genomic and transcriptomic biomarkers—including *FAT1* inactivation (69), *YAP/TAZ* amplification (70), and YAP/TAZ activation gene signatures—can be assessed from tumor biopsies or liquid biopsies to identify tumors with intrinsic pathway dependency. Protein and spatial biomarkers, assessed via multiplex immunohistochemistry, enable simultaneous evaluation of GPCR expression, YAP/TAZ nuclear localization, PD-L1 levels, and the spatial relationships between immune cells and tumor or stromal compartments, thereby providing critical context regarding pathway activity and the mechanisms underlying immune exclusion. Complementing these tissue-based approaches, liquid biopsy and dynamic monitoring strategies—such as circulating tumor DNA (ctDNA) analysis for pathway-relevant mutations, detection of exosomal GPCR and YAP cargo, and peripheral immune cell phenotyping—offer the potential for real-time, non-invasive assessment of pathway engagement and treatment response. Together, these multimodal biomarker approaches will be essential for patient stratification, pharmacodynamic monitoring, and the rational design of combination immunotherapies.

### 5.5 Translational challenges and future directions

Despite the considerable therapeutic promise, clinical translation of GPCR–Hippo–targeting strategies faces several challenges. These include the inherent complexity and redundancy of GPCR signaling, potential tissue-selective toxicities versus on-target immune activation, and the need for validated biomarkers that can reliably stratify patients. Addressing these challenges will require a coordinated effort involving biomarker-driven clinical trials, integrated translational endpoints with deep immune profiling, and a continued focus on elucidating the immune-specific consequences of pathway modulation.

## 6 Future perspectives and research priorities

Despite significant progress in delineating the GPCR--Hippo crosstalk in cancer, several critical knowledge gaps hinder the effective clinical translation of this biology. Addressing these questions through focused research efforts will be paramount for developing successful immunotherapeutic strategies.

### 6.1 Future perspectives and research priorities

#### 6.1.1 Deepening the understanding of the complexity of the GPCR-hippo axis in immune regulation

The immunomodulatory function of the GPCR-Hippo axis is profoundly context-dependent, and a key future direction is to unravel the determinants of this specificity. For instance, why do similar GPCR ligands or YAP/TAZ activation states lead to dichotomous immune outcomes in different tumor types or even in different regions of the same tumor? Answering this will require a deeper investigation beyond tumor cell-intrinsic signaling. A crucial, yet underexplored, area is the intrinsic role of the GPCR-Hippo axis within distinct immune cell subsets. As elegantly reviewed by Tang et al., the non-canonical Hippo pathway, centered on Mst1/2 kinases, is a master regulator of immune homeostasis, governing processes from T cell adhesion and migration to macrophage redox balance and Th17/Treg differentiation (71). Therefore, a priority is to systematically map how GPCR inputs differentially modulate canonical (YAP/TAZ-dependent) versus non-canonical (MST1/2-dependent) Hippo signaling in T cells, macrophages, dendritic cells, and other immune populations within the TME. Such studies will elucidate how these signals intrinsically control immune cell fate decisions, metabolic fitness, and effector functions. Furthermore, a systems-level integration is needed to understand how the GPCR-Hippo network converges with other dominant signals in the TME, including cytokine networks (e.g., TGF-β, IL-6), immune checkpoint pathways, and even microbiome-derived metabolites. Finally, employing spatial multi-omics technologies will be essential to map GPCR and Hippo pathway activity within the architectural context of the TME. This will allow us to correlate signaling states with the spatial organization of immune cells, such as tertiary lymphoid structures or areas of immune exclusion, providing a high-resolution view of the functional crosstalk *in situ*.

#### 6.1.2 Elucidating the molecular basis of tumor type specificity

As highlighted throughout this review, the functional consequences of GPCR-Hippo crosstalk exhibit striking cancer-type specificity. The oncogenic feedback loops (OXTR--YAP in HCC vs. CXCR7--YAP in gastric cancer) and the role of tumor suppressors like GPRC5A in lung cancer underscore this point. A major research priority is to define the underlying genetic and epigenetic landscapes that dictate these tissue-specific responses. This includes understanding how common oncogenic mutations (e.g., in *KRAS*, *TP53*, *AXIN1*) or the specific cellular origin of a tumor shapes the output of the GPCR-Hippo signaling module and its subsequent impact on the immune microenvironment.

### 6.2 Priority areas in translational research

#### 6.2.1 Developing next-generation models with clinical predictive value

To accelerate translation, research must move beyond traditional 2D cell cultures and employ more sophisticated, clinically relevant models. Advanced 3D organoid-immune cell co-culture systems and patient-derived tumor fragment explants that preserve the native TME architecture and cellular heterogeneity are urgently needed. These models will be invaluable for performing high-resolution mechanistic studies and for functionally validating the efficacy of GPCR or Hippo-targeted agents, both as monotherapies and in combination with immunotherapies, in a patient-specific manner.

#### 6.2.2 Targeting core feedback loops in immunotherapy resistance models

A highly promising translational avenue is to directly target the self-reinforcing feedback loops, such as OXTR--YAP in HCC or CXCR7--YAP in gastric cancer, particularly in the setting of acquired resistance to immune checkpoint inhibitors (ICIs). As noted in recent reviews on Hippo pathway targeting (72), overcoming resistance requires strategies that address the adaptive rewiring of signaling networks. Preclinical studies using humanized mouse models or syngeneic models that recapitulate ICI-refractory disease should prioritize testing whether disrupting these loops with specific antagonists (e.g., atosiban for OXTR) can resensitize tumors to ICIs, potentially by dismantling an established immunosuppressive program.

#### 6.2.3 Integrating multi-omics and artificial intelligence to advance precision medicine

The complexity of the GPCR-Hippo-immune network necessitates a multi-disciplinary approach. Future studies should be designed to integrate single-cell RNA-sequencing, spatial proteomics, and functional genomics (e.g., *in vivo* CRISPR screens in tumor-immune co-cultures) to identify novel synthetic lethal interactions or genes that enhance antitumor immunity when the GPCR-Hippo axis is perturbed. Furthermore, the application of artificial intelligence (AI) and machine learning will be critical. AI-driven platforms can be leveraged to analyze complex multi-omic datasets to predict patient-specific responses to GPCR-Hippo targeted therapies, forecast potential immune-related adverse events, and rationally design optimal combination regimens. This integrative approach will be essential for realizing the full potential of precision immunotherapy targeting this central regulatory hub.

## 7 Conclusion

The crosstalk between GPCR signaling and the Hippo pathway constitutes a dynamic regulatory network that profoundly influences cancer biology and antitumor immunity. Its features—G protein subtype-dependent regulation, oncogenic feedback loops, and integration with immune checkpoints—present a diverse array of therapeutic targets. This axis functions as a central hub coordinating tumor--stromal--immune crosstalk, making it a compelling target for immuno-oncology combinations. While challenges exist, interdisciplinary approaches combining novel technologies, drug repurposing, biomarker-guided trials, and a focus on the immune-specific consequences of pathway modulation hold significant promise. Sustained investigation into disrupting core oncogenic-immune feedback loops and understanding spatial specificity within the TME will be pivotal for translating these insights into effective therapies.
